# Potential advantages of FDG‐PET radiomic feature map for target volume delineation in lung cancer radiotherapy

**DOI:** 10.1002/acm2.13696

**Published:** 2022-06-14

**Authors:** Zahra Falahatpour, Parham Geramifar, Seyed Rabie Mahdavi, Hamid Abdollahi, Yazdan Salimi, Alireza Nikoofar, Mohammad Reza Ay

**Affiliations:** ^1^ Department of Medical Physics Tehran University of Medical Sciences Tehran Iran; ^2^ Research Center for Nuclear Medicine Shariati Hospital Tehran University of Medical Sciences Tehran Iran; ^3^ Department of Medical Physics Faculty of Medical Sciences Iran University of Medical Sciences Tehran Iran; ^4^ Department of Radiology Technology Faculty of Allied Medicine Kerman University of Medical Sciences Kerman Iran; ^5^ Department of Biomedical Engineering and Medical Physics Shahid Beheshti University of Medical Sciences Tehran Iran; ^6^ Department of Radiation Oncology Faculty of Medical Sciences Iran University of Medical Sciences Tehran Iran

**Keywords:** grey‐level co‐occurrence matrix, non‐small cell lung cancer, positron emission tomography/ computed tomography, radiomics, radiotherapy, segmentation

## Abstract

**Purpose:**

To investigate the potential benefits of FDG PET radiomic feature maps (RFMs) for target delineation in non‐small cell lung cancer (NSCLC) radiotherapy.

**Methods:**

Thirty‐two NSCLC patients undergoing FDG PET/CT imaging were included. For each patient, nine grey‐level co‐occurrence matrix (GLCM) RFMs were generated. gross target volume (GTV) and clinical target volume (CTV) were contoured on CT (GTV_CT_, CTV_CT_), PET (GTV_PET40_, CTV_PET40_), and RFMs (GTV_RFM_, CTV_RFM_,). Intratumoral heterogeneity areas were segmented as GTV_PET50‐Boost_ and radiomic boost target volume (RTV_Boost_) on PET and RFMs, respectively. GTV_CT_ in homogenous tumors and GTV_PET40_ in heterogeneous tumors were considered as GTV_gold standard_ (GTV_GS_). One‐way analysis of variance was conducted to determine the threshold that finds the best conformity for GTV_RFM_ with GTV_GS_. Dice similarity coefficient (DSC) and mean absolute percent error (MAPE) were calculated. Linear regression analysis was employed to report the correlations between the gold standard and RFM‐derived target volumes.

**Results:**

Entropy, contrast, and Haralick correlation (H‐correlation) were selected for tumor segmentation. The threshold values of 80%, 50%, and 10% have the best conformity of GTV_RFM‐entropy_, GTV_RFM‐contrast_, and GTV_RFM‐H‐correlation_ with GTV_GS_, respectively. The linear regression results showed a positive correlation between GTV_GS_ and GTV_RFM‐entropy_ (*r* = 0.98, *p* < 0.001), between GTV_GS_ and GTV_RFM‐contrast_ (*r* = 0.93, *p* < 0.001), and between GTV_GS_ and GTV_RFM‐H‐correlation_ (*r* = 0.91, *p* < 0.001). The average threshold values of 45% and 15% were resulted in the best segmentation matching between CTV_RFM‐entropy_ and CTV_RFM‐contrast_ with CTV_GS_, respectively. Moreover, we used RFM to determine RTV_Boost_ in the heterogeneous tumors. Comparison of RTV_Boost_ with GTV_PET50‐Boost_ MAPE showed the volume error differences of 31.7%, 36%, and 34.7% in RTV_Boost‐entropy_, RTV_Boost‐contrast_, and RTV_Boost‐H‐correlation_, respectively.

**Conclusions:**

FDG PET‐based radiomics features in NSCLC demonstrated a promising potential for decision support in radiotherapy, helping radiation oncologists delineate tumors and generate accurate segmentation for heterogeneous region of tumors.

## 1 INTRODUCTION

Lung cancer is the leading cause of cancer‐related mortality worldwide and non‐small cell lung cancer (NSCLC) is the most common lung malignancy.^1^, ^2^ Radiotherapy is ordinarily considered as the main treatment option for inoperable NSCLC tumors.^3^ Local tumor recurrence remains the main cause of radiotherapy failure, and caused by intratumoral heterogeneity induced radiotherapy resistance.^4^ Intratumoral heterogeneity refers to the differences within the tumor and provides vital information for the clinical prognosis, and personalized treatment of cancer patients.^5^ Thus an accurate delineation of tumor volume and intratumoral heterogeneity can potentially increase the efficacy of radiotherapy by dose escalation of the heterogeneous areas.^6^, ^7^ In modern radiotherapy, dose escalation can be applied to administer tailored booster doses to heterogeneous areas using techniques such as intensity‐modulated radiation therapy (IMRT) or volumetric modulated arc therapy (VMAT), and also help the patient's treatment response.^8^

To improve the delineation of target volume, fluorodeoxyglucose (FDG) positron emission tomography (PET) has been combined with treatment planning computed tomography (CT).^2,^ ^7^ Using PET/CT images, the metabolic target volume can be segmented with high accuracy.^1^, ^2^ PET/CT fusion empowers physicians with combined anatomical and biological information about tumor, as well as the biological heterogeneity of tumor such as radio‐resistance cells and hypoxia.^9^ Most often, histogram‐based variables like standardized uptake value (SUV) and highest voxel value (SUV_max_) within the region of interest (ROI) are used to target delineation.^2^ SUV_max_ focuses on a single voxel value within the ROI, and thus, depends strongly on noise and cannot aid intratumoral heterogeneity segmentation. Hence, it is not accurate in highly heterogeneous tissues.^10^

Radiomics has become popular in recent years as a way to fully utilize the quantitative data embedded in medical images that a physician's eyes lose through qualitative or semi‐quantitative analysis. Radiomics may be able to show texture features of the image that can only be detected through pathology.^11^ In radiomics, advanced mathematical algorithms are used to extract image features.^12^ Radiomics features are categorized into first‐, second‐, and higher‐order features. First‐order features reflect voxel intensity distribution and include histogram variables, skewness, kurtosis, and distribution variance. Second‐ and higher‐order features reflect the spatial arrangement of voxel values computed from textural matrices such as grey‐level co‐occurrence matrix (GLCM), grey‐level run length matrix (GLRLM), and gray level size zone matrix (GLSZM). For example, GLCM indicates the probability of observing a pair of values in voxels at a specific distance in a specific direction.^12^ It seems that second‐ and higher‐order features can describe tumor heterogeneity better than the first‐order features.^2^

As heterogeneities within the tumors are main causes of radiotherapy failure and different approaches are proposed to personalize the dose based on the heterogeneities,^13,^ ^14^ artificial intelligence including deep learning networks are considered as feasible approaches for therapy volume definition.^15^ For lung cancer radiotherapy, as reviewed by Liu et al.,^16^ several deep networks such as convolutional neural networks (CNNs), fully convolutional networks (FCNs), and generative adversarial network (GAN) were used for both normal tissues and tumor segmentation and a high performance (e.g., sensitivity more than 0.95) is obtained by these methods.

The texture features have been used for target definition in several studies. In a study by Markel et al.,^17^ combination of PET and CT texture features and K‐nearest neighbors (KNN) classifier was used for gross tumor volume delineation in lung carcinoma patients and sensitivity of 73.9% was obtained. Liu et al.,^18^ applied PET/CT texture features for the recognition of tumors and organs at risk for radiotherapy treatment planning. They proposed biological target volume, based on PET features including busyness, contrast, as well as SUV by a hierarchical Mumford‐Shah Vector Model.

The radiomic target volume is also suggested as a new radiotherapy volume which reflects tumor heterogeneities.^19^ Johanian et al. assessed the ability of texture features for better delineation of malignant tissue in FDG‐PET images of lung cancer. They found that textural parameters seem appropriate to differentiate tumoral tissue from normal lung tissue.^20^ Furthermore, Yu et al.^7^ developed a co‐registered multimodality pattern analysis segmentation system (COMPASS) by using PET and CT texture analysis for volume contouring in head and neck cancer patients and results were compared to radiation oncologists contouring.

In the current study, we aimed at examining the suitability of PET radiomic feature maps (RFMs) derived from GLCM texture features for target definition in radiotherapy planning of lung cancer patients. In this work, heterogeneities revealed by PET RFMs as well as contribution of radiation oncologists to decisions about these target volumes were studied.

## 2 MATERIALS AND METHODS

### 2.1 Patient characteristics

Thirty‐two NSCLC patients undergoing FDG PET/CT imaging were included. As summarized in Table 1, they included 24 men and 8 women with a mean age of 65 ± 9.1 years. The inclusion criteria were histopathologically proven stage II or III NSCLC cases. Patients with stage I or IV were excluded, as stage I may not require radiotherapy and Stage IV, in some cases, contains diffused tumor volume that may reduce contouring accuracy. The patients were deemed inoperable by the surgeons, and a radiation oncologist consulted the research team during the study. The Institutional Review Board approved the study, and all methods were performed in accordance with the relevant guidelines and regulations.

### 2.2 PET/CT acquisition

All the patients were requested to follow a high‐protein, low‐carbohydrate diet to reduce myocardial (FDG) uptake in PET imaging. They were instructed to fast for six hours prior to FDG injection. One‐hour post‐injection low dose CT scan was performed followed by a whole‐body PET scan. The total scanning time was about 30 min. Reconstruction matrix size on CT images was 512 × 512 for each trans‐axial slice with a voxel size of 2 × 2 × 3 mm^3^. In PET images, the reconstruction matrix size of trans‐axial slices was 168 × 168 with 4 × 4 × 3 mm^3^ voxel size. The PET images were co‐registered with CT images. For merging the CT and PET images, they were aligned and resampled using an established registration toolbox for transformation. The toolbox is syngo application software (Siemens healthcare, Erlangen, Germany) with rigid registration capability for viewing images from various digital imaging procedures.

### 2.3 Radiomic feature mapping

All feature mapping were performed in the Matlab R 2018b program using an adapted version of the Computational Environment for Radiotherapy Research (CERR).^21^ The PET images were first imported into CERR; feature values were extracted and converted into “feature maps.” All GLCM‐related features, including the nine features of entropy, contrast, correlation, Haralick‐correlation (H‐correlation), homogeneity, energy, cluster shade, cluster prominence, and sum average (Table S1).^22^, ^23^ were extracted from the entire image. Then their feature maps were saved in “.mat” format. The representation of these textures was based on their implementation in CERR. The extracted feature maps were converted into DICOM format images using in‐house software to be matched with TPS (Figure S1).

### 2.4 Gross target volume delineation using CT images (GTV_CT_)

Two experienced radiation oncologists and two nuclear medicine physicians, being unaware of the patients’ diagnosis or history, defined the FDG uptake heterogeneity in the PET images. The decision was made visually by a scoring system; each expert evaluated the tumor and assigned a score of: zero for the homogeneous uptake and one for the heterogeneous uptake. Tumors with the average score above three were selected as a heterogeneous tumor.^24,^ ^25^ CT images, PET images, and feature maps were all fused together in the RayStation treatment planning system (TPS; RaySearch Laboratories AB, Stockholm, Sweden).^26^ First, the gross target volume (GTV) was delineated manually on the CT images of all 32 patients without knowledge of the PET information. Tumor delineation was performed through consultation with two radiation oncologists having more than 10 years of experience. GTV_CT_ was contoured using a lung window of 1600 HU and level of −300 HU when the GTV located inside the lung tissue, and mediastinal window setting (window, 600 HU; level, 40 HU) was modified when the tumor was close to the mediastinum.^27^ This study only considered the primary tumors and did not include suspicious lymph nodes.

### 2.5 GTV delineation using PET images with 40% of SUVmax (GTV_PET40_)

There are several methods for PET image segmentation, including: manual segmentation and ground truth reconstruction, stochastic and learning‐based, thresholding‐based, region‐based, boundary‐based, and multi‐modality methods.^28^ In this study, GTV_PET_ was determined using the threshold‐based contouring method. Several studies have accepted 30%–75% of SUV_max_ as a threshold for delineating the tumor area in lung cancers.^7^, ^12^, ^29^ However, the application of 40% threshold is more common in other studies.^30^, ^31^, ^32^ Therefore, the same threshold value was used in the current study. Consequently, GTV_PET40_ was delineated as a tumor area on the PET images for all patients.

### 2.6 GTV_Gold standard_ (GTV_GS_)

**FIGURE 1:**
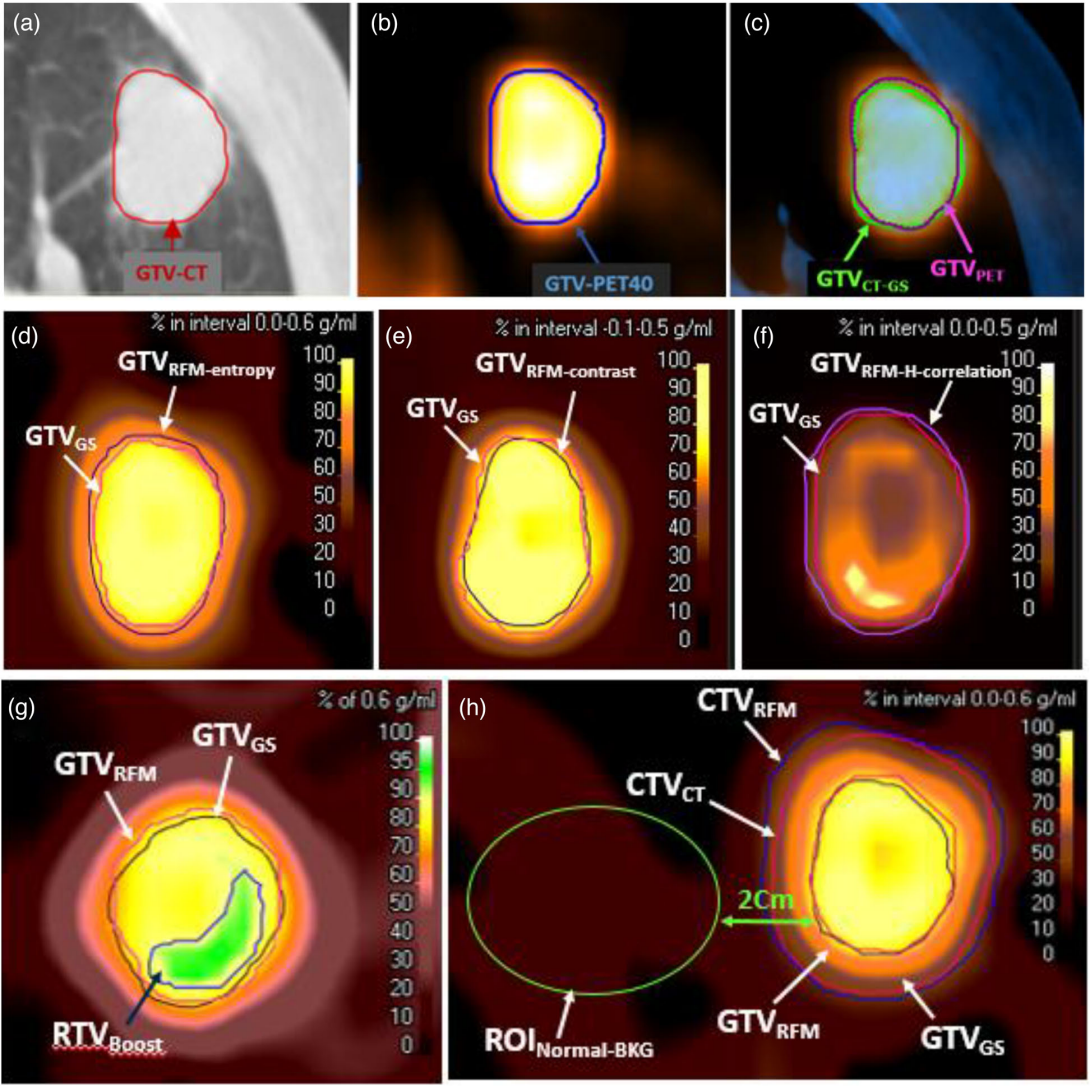
(a) GTV was contoured manually in CT images, (b) GTV was contoured by a threshold of 40% in PET image, and (c) fusion GTV_CT_ and GTV_PET_ as a GTV_GS._ The tumor volume in the axial view in (d) entropy feature map, (e) contrast feature map and (f) H‐correlation feature map of a patient image. (g) RTV_Boost_ and GTV_RFM_ contours in a heterogeneous tumor. The dark blue line represents the threshold of 90% of the maximum entropy values, as a RTV_Boost‐entropy_. (h) CTV_CT_ and CTV_RFM_ contours in the entropy feature map. CTV_CT_ was created by adding a fixed margin of 8 mm while fixed threshold of the 45% of maximum entropy feature value was used for defining the CTV_RFM_

We determined the gold standard GTV (GTV_GS_) in the previously defined homogenous tumors. In these cases, tumor volumes in the CT and PET images were not significantly different because of less likely necrosis in the small tumors^33^ (as shown in Figure 1c). Hence, GTV_CT_ was considered GTV_GS_ and PET images were registered on the CT images in order to check the accuracy of delineation.

### 2.7 GTV delineation using PET RFM (GTV_RFM_)

Nine GLCM feature maps were generated for all the patients. A panel of four experts, including two oncologists and two nuclear medicine physicians selected the feature maps based on i) image quality, ii) distinguishability from surrounding tissue, and iii) edge clearness. Each expert evaluated the feature maps and assigned a value for the accepted items and zero for the rejected items based on the criteria mentioned above. Feature maps with the score above three were selected. Then they were used to delineate the tumor volume by the thresholding method (Figure 1d–f). In particular, we tried various threshold percentages on the feature maps and compared the obtained tumor volume with GTV_GS_ to get the best match as a GTV_RFM_. Dice similarity coefficient (DSC) was used to assess the similarity between GTV_RFM_ and GTV_GS_ in homogeneous tumor, because it is one of the most commonly used quantitative metrics for evaluating segmentation accuracy. It measures spatial overlap between a segmented tumor and the surrogate truth (in percentage). DSC has a range of [0, 1]. It is recommended that a good overlap occurs when DSC > 0.700.^34^ DSC was calculated as follows:

(1) $$\begin{array}{ccc} & & \mathrm{DSC}\;\;\;\left(\mathrm{GT}V_{\mathrm{GS}},\;\mathrm{GT}V_{\mathrm{RFM}}\right)\\ & & =\;2\;\times \;\frac{|\;\mathrm{GTVGS}\cap \mathrm{GTV}{\;}_{\mathrm{RFM}}|\;}{\mathrm{GT}V_{\mathrm{GS}}\;+\;\mathrm{GTV}{\;}_{\mathrm{RFM}}}\times 100\; \end{array}$$

### 2.8 Intra‐tumoral heterogeneity delineation using RFM (RTV_Boost_)

The selected feature maps could reveal regions with dissimilar FDG uptake. Higher values of entropy are associated with higher intra‐tumoral heterogeneity.^24,^ ^35^ Figure 1g, shows an example of intra‐tumoral segmentation using an entropy feature map. The maximum entropy values are depicted as a pink region. To determine radiomic boost target volume (RTV_Boost_) in heterogeneous tumors, two radiotherapy oncologists defined a threshold level that generates an area with minimum feature value greater than the average value of GTV_RFM_. The subregional heterogeneity was named as RTV_Boost_, was shown in Figure 1h. RTV_Boost_ in the selected feature maps was also compared with RTV_Boost_ (50% of SUV_max_) proposed as the optimum threshold for delineating the PET intra‐tumoral heterogeneity for dose escalation.^9^, ^36^, ^37^ To assess the volume differences between RTV_Boost_ and GTV_PET50‐Boost_, the mean absolute percent error (MAPE) was used.^38^ The following equation was used to assess the volume differences between GTV_PET50‐Boost_ and RTV_Boost_ (in percentage):

(2) $$\begin{array}{ccc} & & \mathrm{MAPE}\;\;\left(\mathrm{GTVPET}50-\mathrm{Boost},\;\mathrm{RTVBoost}\right)\\ & & =\frac{1}{n}\sum_{1}^{n}\frac{|\;\mathrm{RTVBoost}\;-\mathrm{GTVPET}50-\mathrm{Boost}|}{\;\mathrm{GTV}\;\mathrm{PET}50-\mathrm{Boost}}\;\times 100 \end{array}$$

where, *n* is the number of patients.

### 2.9 Clinical target volume delineation using PET RFM (CTV_RFM_)

clinical target volume (CTV) was created by extending the GTV margin (e.g., 6–8 mm) to include any possible microscopic cells around the primary tumor in the CT images. The GTV margin was added uniformly to the GTV edges in all directions.^39^ In this study, an average of 8 mm margin was applied around the GTV_CT_ to create the CTV_CT_. The feature maps mentioned in previous section were used to determine a reasonable threshold for contouring of the CTV_RFM_. For this purpose, by consulting two expert radiation oncologists, the ROI was identified in the normal background tissue 2 cm away from the edges of GTV with the same volume as the tumor (ROI _Normal‐BKG_). We analyzed various threshold values; the value that generated concentrations greater than 1.5 times the maximum value of the feature in ROI _Normal‐BKG_ was considered as CTV_RFM_ (Figure 1h). The MAPE was used to assess the volume differences between CTV_RFM_ and CTV_GS_.

### 2.10 Statistical analysis

GraphPad Prism software (ver. 8.4.3) was applied to determine the threshold that finds the best conformity for GTV_RFM_ with GTV_GS._ DSC and the MAPE were used to assess the similarities and differences between the volumes, respectively. In addition, linear regression analysis (LRA) was used to report the correlations between the volumes of GTV_GS_ and GTV_RFM_, CTV_GS_ and CTV_RFM_, and RTV_Boost_ and GTV _PET50‐Boost._ According to our assumptions, there exists a relationship between volumes in each group and any variations in one volume are responsible for causing the variation in the other. Moreover, LRA is a suitable statistical method for calculating p‐values and finds the line that most closely fits the GTV_RFM_ (or CTV_RFM_ and RTV_Boost_) on GTV_GS_ (or CTV_GS_ and GTV_PET50‐Boost_) according to a specific mathematical criterion. P‐values less than or equal to 0.05 were considered as statistically significant.

## 3 RESULTS

**FIGURE 2:**
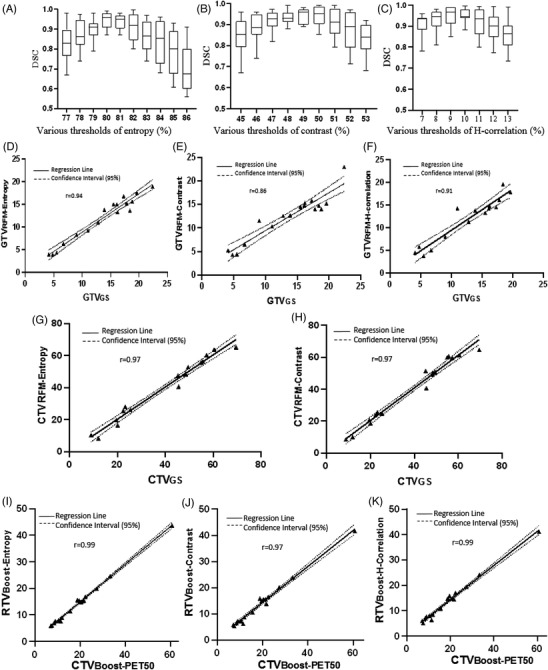
Box plot of DSC using various thresholds of: (a) entropy (b) contrast, and (c) H‐correlation. Error bars indicate standard deviation. Second row; LRA: GTV_GS_ versus (d) GTV_RFM‐entropy_, (e) GTV_RFM‐contrast_, (f) GTV_RFM‐H‐correlation_ in the homogeneous tumors. LRA: CTV_GS_ versus (g) CTV_RFM‐entropy_, (h) CTV_RFM‐contrast_ in the homogeneous tumors. LRA: GTV_PET50‐Boost_ versus (i) RTV_Boost‐entropy_, (j) RTV_Boost‐contrast_, and (k) RTV_Boost‐H‐correlation‐_ in the heterogeneous tumors

NSCLC tumors were segmented on CT and PET images of 32 patients. The maximum diameter of GTV_CT_ was 6.5 cm, and the tumor volume was 45.6 ± 71.0 cm^3^. These values were smaller for GTV_PET_ in all the patients, that is, 5.7 cm and 17.6 ± 19.3 cm^3^, respectively. As presented in Table 2, we ultimately selected three feature maps with scores above three for GTV_RFM_ contouring. The selected features were entropy, contrast, and H‐correlation, where GTV_RFM_ was contoured in this benchmark group using the threshold tools on the selected feature maps (Figure 1–f). The aforementioned visual scoring system resulted in 17 cases of homogenous tumors among 32 cases. Most of the tumors with diameter smaller than 3 cm were categorized in the group of homogenous tumors. In this group, GTV_CT_ was identified as GTV_GS_ (Table 3). In particular, we tested various threshold percentages on entropy, contrast, and H‐correlation feature maps to determine GTV_RFM_ (Figure 2–c).

**TABLE 1:** Patient demographic data

**Patient characteristics**	**Number (%)**
**Gender**	
Male	24 (75.0)
Female	8 (25.0)
**Age (year)**	
Median (range)	65 (41‐79)
**Location**	
Right upper lobe	10 (31.2)
Right lower lobe	7 (21.8)
Left upper lobe	5 (15.6)
Left middle lobe	6 (18.7.)
Left lower lobe	4 (1.5)
**TNM classification (%)**	
T2N0M0	8 (25.0)
T3N0M0	17 (53.1)
T2N1M0	7 (21.8)

**TABLE 2:** Feature selection based on visual characteristics. Features with score above 3 were selected (bolded features)

		**Selection factors**				
		**Visual characteristics**				
	**GLCM feature maps extracted from CERR**	**Experts scores**				
**No**		1	2	3	4	**Result**
1	**Entropy**	1	1	1	1	4
2	**Contrast**	1	1	1	1	4
3	**Haralick correlation**	1	1	1	1	4
4	Local homogeneity	1	0	0	1	2
5	Energy	1	1	0	0	2
6	Correlation	1	0	0	0	1
7	Cluster shade	1	1	0	0	2
8	Cluster prominence	1	1	0	0	2
9	Sum avg	1	0	0	1	2

**TABLE 3:** Comparison of volumes in GTV_GS_, GTV_PET40_, and GTV_RFM_ by DSC in the homogeneous tumors

	**GTV_GS_ (GTV_CT_**)		**GTV_PET40_**	**GTV_RFM‐entropy = 80%_**	**DSC for volume comparison GTV_GS_ vs. GTV_RFM‐entropy_ in the threshold of 80%**	GTV_RFM‐contrast = 50%_	**DSC for volume comparison GTV_GS_ vs. GTV_RFM‐contrast_ in the threshold of 50%**	GTV_RFM‐H‐correlation = 10%_	**DSC for volume comparison GTV_GS_ vs. GTV_RFM‐H‐correlation_ in the threshold of 10%**
**Case**	Size (cm)	Volume (cm^3^)	Volume (cm^3^)	Volume (cm^3^)	Avg = 0.92	Volume (cm^3^)	Avg = 0.92	Volume (cm^3^)	Avg = 0.91
1	2.9	19.6	17.2	17.6	0.94	15.2	0.87	17.9	0.95
2	2.3	16.1	14.6	15	0.96	14.8	0.95	14.3	0.94
3	2.5	17.3	16.3	16.8	0.98	15.8	0.95	14.5	0.91
4	3	18.4	15.7	13.6	0.85	14.8	0.89	19.6	0.97
5	2.7	17.8	15.9	15.2	0.92	14	0.88	16.1	0.95
6	2.4	13.9	11.9	13.8	0.89	12.7	0.95	13.8	0.89
7	2.2	4.1	3.1	3.9	0.96	5.2	0.88	4.5	0.94
8	2.4	9	7.9	8.2	0.95	11.5	0.87	8.1	0.95
9	2.1	4.8	3.8	3.9	0.89	4.3	0.95	5.8	0.89
10	2.3	5.5	4.2	4.3	0.88	4.4	0.82	3.8	0.82
11	2.5	12.8	10.5	11	0.92	12.6	0.99	11.3	0.94
12	2.4	16.2	15.1	13.3	0.90	15.3	0.97	14.8	0.95
13	2.5	6.7	6.2	6.3	0.97	6.5	0.98	5.1	0.86
14	2.6	15.5	13.9	15.0	0.98	14.5	0.96	13.3	0.92
15	2.2	11	10.2	9.2	0.91	10.3	0.96	14.3	0.87
16	2.8	18.8	13.9	15.6	0.91	14	0.85	13.5	0.83
17	2.9	22.4	15.1	18.9	0.89	24.9	0.94	20	0.94

The threshold value for the best matching of GTV_RFM‐entropy_ and GTV_GS_ was achieved in 80% of the maximum entropy value with the average DSC value of 0.93. Various threshold percentages were also tested for the contrast and H‐correlation feature maps in the benchmark group. The best matching values were achieved at the 50% threshold of maximum contrast values with average DSC value of 0.92. For H‐correlation feature map, the best matching volume was achieved at the threshold of 10% with the average DSC of 0.91 (Table 3). The linear regression results showed a positive correlation between GTV_GS_ and GTV_RFM‐entropy_ (*r* = 0.98, *p* < 0.001), between GTV_GS_ and GTV_RFM‐contrast_ (*r* = 0.93, *p* < 0.001), and between GTV_GS_ and GTV_RFM‐H‐correlation_ (*r* = 0.91, *p* < 0.001) (Figure 2d–f).

As mentioned earlier, we used the feature maps to determine CTV_RFM_. In the homogeneous tumors, the average threshold values of 45% and 15% were obtained for contouring CTV_RFM‐entropy_ and CTV_RFM‐contrast_, respectively. CTV_RFM‐entropy_ and CTV_RFM‐contrast_ were compared with the CTV_CT_ MAPE values of 5.0% and 6.2%, respectively (Table S3). The linear regression outcome showed a positive correlation between CTV_GS_ and CTV_RFM‐entropy_ (*r* = 0.97, *p* < 0.001) and between CTV_GS_ and CTV_RFM‐contrast_ (*r* = 0.96, *p* < 0.001) (Figure 2,h).

After ascertaining the threshold percentages mentioned above in the homogeneous tumors group, the obtained threshold values were generalized and evaluated in the heterogeneous tumors to determine GTV_RFM_. In these patients GTV_PET40_ was considered as a GTV_GS_ and MAPE was used to show the differences in volumes of GTV_PET40,_ GTV_RFM‐entropy_, GTV_RFM‐contrast,_ and GTV_RFM‐H‐correlation_. As presented in Table S2, in comparing GTV_RFM_ with GTV_GS_ or GTV_PET40_, MAPE values showed volume error differences of 10.5%, 11.1%, and 10.5% in GTV_RFM‐entropy_, GTV_RFM‐contrast,_ and GTV_RFM‐H‐correlation_, respectively.

Furthermore, to delineate the microscopic extension cells around the gross tumor in heterogeneous tumors, CTV_RFM_ was segmented using the thresholds mentioned above in homogeneous case. The volume difference between CTV_RFM_ and CTV_PET_ was calculated by MAPE and the result is presented in Table S3. Comparison of CTV_RFM_ with CTV_PET_ MAPE, showed the volume error differences of 10.5% and 9.4% in CTV_RFM‐entropy_ and CTV_RFM‐contrast,_ respectively (Table S4).

Moreover, we used the feature maps to determine RTV_Boost_ (intra‐tumoral heterogeneity) in the heterogeneous tumors, and the threshold percentage was obtained in 90%, 55%, and 33% in the entropy, contrast, and h‐correlation feature maps, respectively. The linear regression results revealed a positive correlation between GTV_PET50‐Boost_ and RTV_Boost‐entropy_ (*r* = 0.99, *p* < 0.001), between GTV_PET50‐Boost_ and RTV_Boost‐contrast_ (*r* = 0.97, *p* < 0.001), and between GTV_PET50‐Boost_ and RTV_Boost‐H‐correlation_ (*r* = 0.99, *p* < 0.001) (Figure 2i–k).

## 4 DISCUSSION

Accurate target definition based on intra‐tumoral heterogeneity is a critical issue in radiation therapy. Tumor heterogeneity is a main prognostic factor for radiation therapy outcome and as is connected to higher recurrence risk.^40,^ ^41^ Recently, the potential of radiomics on the assessment of tumor heterogeneity and improvement of personalized medicine has been highlighted.^42^ It is clarified that PET image textural features may have more effective role rather than conventional uptake factors, like SUV_max_ for several clinical purposes.^40^, ^41^ In the current research, suitability of second‐order PET features (calculated based on GLCMs) for accurate identification of the tumor volume and delineation of intra‐tumoral heterogeneity of NSCLC was examined. PET GLCM feature maps were converted into DICOM format, imported and registered in TPS alongside patient's PET and CT images. This empowers radiation oncologists to become familiar with RFM heterogeneities underneath PET images, which were not easily detected in labor‐intensive tumor volumes delineation processes. Thus, precise and consistent delineation of tumor volumes as well as contribution of radiation oncologists to decisions about the target volumes were the superiority of our study.

In our approach, we divided subjects into two groups including patients with homogenous and heterogeneous tumors. We observed that there was no significant difference in tumor volume between the CT and PET images in the homogeneous tumors in accordance with other investigations.^24,^ ^25^ Therefore, we considered GTV_CT_ as a GTV_GS_ in this group of patients. To determine GTV_RFM_ in feature maps, by testing different threshold levels and comparing the generated tumor volume with GTV_GS_ via DSC (Table 3), we found that the threshold levels of 80%, 50%, and 10% of maximum feature value could accurately contour the target volume in the entropy, contrast, and H‐correlation feature maps, respectively. According to Table 3, PET image contours underestimate the tumor volume; GTV_PET40_ and GTV_RFM_ showed slight differences with GTV_GS_ (GTV_CT_) in the homogeneous tumors, while GTV_RFM_ was closer to GTV_GS_ than GTV_PET40_. It is to be noted that, underestimation is less noticeable when GTV_RFM_ is compared with GTV_PET40_, and gets more significant when GTV_RFM_ is compared with higher values of threshold like GTV_PET50_ applied for tumor contouring.^43^, ^44^, ^45^, ^46^ Our findings are consistent with those of Hatt et al.,^28^ who evaluated several methods of tumor‐contouring algorithms in PET imaging (e.g. fixed thresholds with 40% and 50% of SUV_max_, region‐based, clustering, and statistical methods) and reported that in fixed threshold methods, 40% of SUV_max_ was superior to 50% of SUV_max_.

Moreover, in the group of heterogeneous tumors (Table S2), a significant difference was observed between GTV_PET40_ and GTV_RFM_ with the MAPE values of 10.5%, 11.1%, and 10.5% for entropy, contrast, and H‐correlation, respectively. It seems that the presence of more heterogeneous regions in larger tumors decrease the accuracy of tumor volume contouring in PET images; this finding is in accordance with the findings of other studies.^41,^ ^47^ This may be related to the fact that SUV_max_ cannot identify the total activity of the whole tumor in PET images, because a single voxel may not explain the overall uptake heterogeneity in the entire tumor.^24^ Meanwhile, a second order‐based GLCM feature provides more information than SUV_max_ about the spatial relationship of image voxels. Also, the surface scheme of GLCM represents the spatial intensity distribution, which is, generally, undetermined in first‐order histogram analyses.^41^ Hence, when determining the tumor volume on PET/CT images in radiotherapy, RFMs have a good potential to consider instead of PET images, as they provide actual radiobiological maps.

Besides tumor boundary delineation, evaluation of intra‐tumoral heterogeneity in PET images has become an interesting research topic.^24^ Hatt et al. applied the fuzzy locally adaptive Bayesian (FLAB) technique for heterogeneity segmentation of tumors in PET images, considering its three‐class intra‐tumoral segmentation capacities.^48^ Moreover, Soufi et al. proposed a new framework for automated segmentation of homogeneous and heterogeneous lung tumors in FDG‐PET imaging. They used a novel fuzzy random walk algorithm, which showed a significantly improved performance relative to conventional random walk segmentation.^6^ In the present study, we utilized PET feature maps to segment highly heterogeneous intra‐tumoral regions quantitatively. In accordance with other studies, entropy, as the most popular textural feature in local heterogeneity tumor studies, measures the intra‐tumoral heterogeneity relative to changes in the FDG uptake between voxels.^24^, ^41^, ^49^ We found the entropy feature map a robust feature as due to its highest observed score (Table 2) with high DSC value of 0.93 (Table 3) and significant potential to segment intra‐tumoral heterogeneity as RTV_Boost_ (Table 4). Bundschuh et al., proposed a new segmentation algorithm based on textural features in FDG‐PET/CT imaging of lung tumors and reported that entropy feature ensures the most precise tumor contouring.^50^

**TABLE 4:** Comparison of volumes in GTV_RFM‐Boost_ and GTV_PET50_ in the heterogeneous tumors

	GTV_GS_ (GTV_PET‐40)_	GTV_PE T50‐_ _Boost_	GTV_RFM‐_ _entropy‐_ _Boost_	Threshold for GTV_RFM‐_ _entropy‐Boost_	Comparison of GTV_PET50_ and GTV_RFM‐entropy‐_ _Boost_	GTV_RFM‐_ _contrast‐_ _Boost_	Threshold for GTV_RFM‐_ _contrast‐_ _Boost_	Comparison of GTV_PET50_ and GTV_RFM‐contrast‐_ _Boost_	GTV_RFM‐H‐_ _correlation‐Boost_	Threshold for GTV_RFM‐H‐_ _correlation‐Boost_	Comparison of GTV_PET50_ and GTV_RFM‐H‐_ _correlation‐Boost_
**Case**	Volume (cm^3^)	Volume (cm^3^)	Volume (cm^3^)	Avg = 90 (%)	MAPE (%) = 31.7	Volume (cm^3^)	Avg = 55 (%)	MAPE (%) = 36.0	Volume (cm^3^)	Avg = 33(%)	MAPE (%) = 34.7
1	10.6	7.5	5.9	92	27.1	5.3	64	43.3	5.1	33	47
2	42.1	27.4	19.8	88	38.3	20.1	40	36.3	19.3	25	41.9
3	16.2	10.5	7.8	92	38.4	7.2	55	45.8	7.9	36	32.9
4	12.4	9	7.5	90	20	7.1	63	26.7	7.7	37	16.8
5	29.4	20.4	14.8	89	37.8	15.6	62	30.7	14.7	26	38.7
6	30.3	22.4	16.8	92	33.3	16.4	63	36.5	17	31	31.7
7	10.2	7.1	5.8	88	22.4	5.8	56	22.4	6	29	18.3
8	13.9	11.9	8.9	88	33.7	8.7	57	36.7	8.5	32	40
9	22.1	15.6	11.3	90	38	10.5	44	48.5	10.8	27	44.4
10	28	19.6	15.3	88	28.1	14.8	59	32.4	15.8	25	24
11	14.2	8.5	6.9	92	23.1	6.5	61	30.7	7	31	21.4
12	32.5	21.3	15.2	93	40	15.5	60	37.4	14.8	37	43.9
13	25.4	18.8	15.5	88	21.2	15.9	57	18.2	14.8	35	27
14	85.6	60.7	43.7	92	38.9	41.6	55	45.9	41.1	33	47.6
15	50.5	33.3	24.6	90	35.3	23.9	51	39.3	24.1	34	38.1
16	16.7	11.4	7.5	87	34.2	6.2	57	45.6	6.4	29	43.8
17	33.2	21.6	15.3	88	29.1	13.8	51	36.1	14.4	37	33.3

This study also showed that contrast feature maps are valuable for intra‐tumoral segmentation. Generally, contrast measures the gray level or intensity variations between the reference pixel and its neighbors; a high contrast reflects large intensity differences in GLCM.^22,^ ^51^ Contrast is affected by heterogeneity,^52^ and we found it as a potential biomarker in determining intra‐tumoral heterogeneous uptake areas and contouring RTV_Boost_. In the study conducted by Qian Zhao et al., entropy‐ and contrast‐extracted textural features in 379 segmented solitary pulmonary nodules resulted in higher values in malignant regions than in benign regions.^22^ Therefore, we determined intra‐tumoral heterogeneity segmented by contrast index as RTV_Boost_ as (Table 4). As shown in Figure 1f, H‐correlation which measures the linear dependency of gray levels on those of neighboring pixels with the levels run from zero to the maximum gray level minus 1, can delineate intra‐tumoral heterogeneous uptake areas, and thus, determine the RTV_Boost_ through segmentation of the areas with higher values of feature (Table 4). As presented in Table 4, the threshold values of 90%, 55%, and 33% of maximum feature values could identify intra‐tumoral heterogeneity as RTV_Boost_ in entropy, contrast, and H‐correlation feature maps, respectively. However, the boost volumes obtained in this method are smaller than those obtained by threshold of 50% of SUV_max_ reported in other studies.^9^, ^36^, ^37^ It is worth nothing that, the size of boost volume is a crucial factor in the dose escalation procedure.^9^ Smaller boost volumes allow dose escalation to eliminate the radiation‐resistant cells, increase tumor control, and decrease the risk of recurrence without increasing the dose to the surrounding normal organs.^8^, ^29^ Since biological effective dose (BED) escalation above 100 Gy improves the tumor control in all kinds of NSCLC^53^, ^54^ and above 120 Gy increases survival in squamous cell carcinoma (SCC),^53^ larger volumes of boost may make it difficult to deliver higher doses due to the surrounding normal organs. Thus, we suggest utilizing RTV_Boost_ in dose painting or as a “micro‐boost” in the delivery step of BED values above 100 Gy.

We further also investigated the usefulness of feature maps in determining CTV for homogenous (CTV_CT_) and heterogeneous (CTV_PET40_) tumors. As mentioned earlier (Section 2.9), in a radiotherapy routine, CTV_CT_ is delineated by adding an identical margin around the tumor, considering the high potential of microscopic cancer cells surrounding GTV. As shown in Table S3, the threshold levels of 45% and 15% of maximum feature values can help substitute CTV_CT_ in homogenous tumors for entropy, contrast feature maps. The results showed that CTV_RFM_ was beyond or inside the CTV_CT_ border in some boundaries around the homogenous tumors (Table S3). The same finding was observed for heterogeneous tumors, as shown in Table S4. These findings are in accordance with the results of Loon D et al., who demonstrated that microscopic disease extension around the tumor in pathological examinations (CTV_path_) is not distributed uniformly around GTV.^39^ CTV_RFM_ has more potential than CTV_CT_ and CTV_PET_ because feature maps inherently extracted from the spatial‐intensity distribution of second‐order features are usually masked by first‐order features such as volume in CT or SUV_max_ in PET images. It should be noted, we could not find a suitable threshold for contouring CTV_RFM‐H‐correlation_ because the threshold value that determined the CTV_RFM‐H‐correlation_ was unrepeatable in different patients.

## 5 CONCLUSION

The present research results revealed the potential advantages of textural features in improving the definition of GTV, CTV, and intratumoral heterogeneity for dose painting in NSCLC as depicted by GTV_RFM_, CTV_RFM_, and RTV_Boost_. While the obtained results are promising, further research is needed to assess and validate their clinical application in practice.

### 5.1 Limitations

The major limitation of the study is the small sample size. However, considering it as a pilot study, the obtained results indicate the potential benefit of RFM‐derived heterogeneity in lung cancer radiotherapy, and the current sample size aligns the informational value of this study. Further large‐scale studies are warranted to replicate and extend these findings.

## CONFLICT OF INTEREST

The authors declare that there is no conflict of interest that could be perceived as prejudicing the impartiality of the research reported.

## AUTHOR CONTRIBUTIONS

*Study conception and design*: Parham Geramifar, Seyed Rabie Mahdavi, and Zahra Falahatpour. *Data collection*: Yazdan Salimi and Zahra Falahatpour. *Analysis and interpretation of results*: Parham Geramifar, Alireza Nikoofar, Hamid Abdollahi, and Mohammad Reza Ay. *Draft manuscript preparation*: Zahra Falahatpour and Parham Geramifar. All authors reviewed the results and approved the final version of the manuscript.

## Supporting information

Supporting Information.

## Data Availability

All data generated and analyzed during this study are included in this published article (and its supplementary information files).
