# Supplementary material for: Potential advantages of FDG‐PET radiomic feature map for target volume delineation in lung cancer radiotherapy
Source: J Appl Clin Med Phys. 2022 Jun 14;23(9):e13696. doi: 10.1002/acm2.13696 (PMC9512354; doi:10.1002/acm2.13696)
Supplement: Supplementary file 1 — Supporting Information. [file ACM2-23-e13696-s001.docx]

**Supplementary appendix**

Supplementary to:

Potential Advantages of FDG-PET Radiomic Feature Map for Target Volume Delineation in Lung Cancer Radiotherapy

**Figure S.1.** A same slice of a patient: A) a PET Image, B) an exported GLCM feature map from CERR, C) the feature map that was converted to Dicom format, and D) Dicom feature map that was improved.

**Table S.1.** A list of GLCM texture features extracted from CERR

**Table S.2.** Comparison of volumes in GTV_PET_, GTV_RFM-entropy_, GTV_RFM-contrast_ and GTV_RFM-H-correlation_ in the heterogeneous tumors.

**Table S.3.** Comparison of volumes in CTV_CT_ and CTV_RFM_ by MAPE (%) in the homogeneous tumors.

**Table S.4**. Comparison of volumes in CTV_PET_ and CTV_RFM_ (in entropy and contrast features) by MAPE (%) in the heterogeneous tumors.


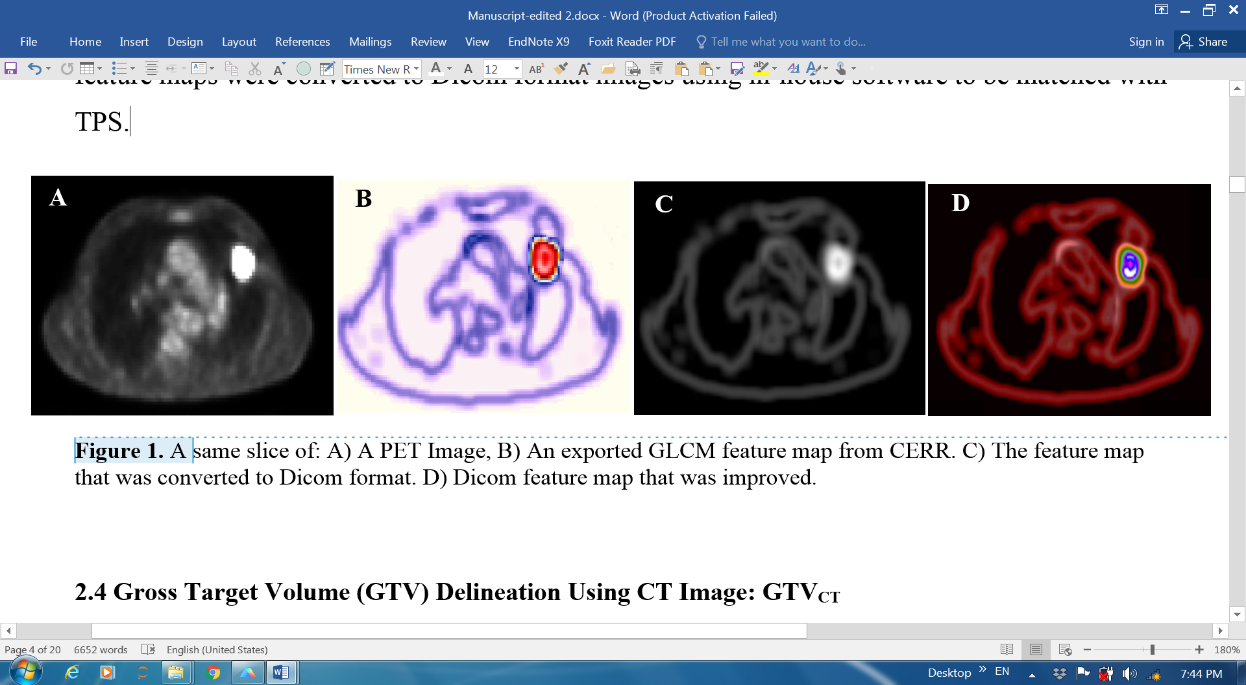
**Figure S.1.** A same slice of a patient: A) a PET Image, B) an exported GLCM feature map from CERR. C) the feature map that was converted to Dicom format, and D) Dicom feature map that was improved.

**Table S.1.** A list of GLCM texture features extracted from CERR

| **GLCM features** | **Description** | **Formula** |
| --- | --- | --- |
| **Entropy** | measures the occurrence of random pair of pixels | $\sum_{i,j} {g\left( i,j \right)log}_{2}g\left( i,j \right)$ |
| **Contrast** | measures the gray level or intensity variations between the reference pixel and its neighbors | $\sum_{i,j} {g\left( i-j \right)^{2}log}_{2}g\left( i,j \right)$ |
| **Correlation** | Measures the linear dependency of a pair of pixels in the image | $\sum_{i,j} \frac{\left( i-\mu\right)\left( j-\mu\right)g \left( i,j \right)}{\sigma^{2}}$ |
| **H-Correlation** | Measures the linear dependency of gray levels on those of neighboring pixels with the levels run from 0 to the maximum gray level minus 1 | $\sum_{i,j} \frac{\left( i,j \right)g \left( i,j \right)-\mu^{2}t}{\sigma^{2}}$ |
| **Homogeneity** | measures image homogeneity with larger values for smaller gray tone difference in pair object. | $\sum_{i,j} \frac{\left( i-\mu\right)\left( j-\mu\right)}{1+\left( i-j \right)^{2}}g \left( i,j \right)$ |
| **Energy** | measures the degree of pixel pair repetitions. It is the measurement of disorder in texture in an image | $\sum_{i,j} g\left( i,j \right)^{2}$ |
| **Cluster shade** | measures skewness of the GLCM matrix, and it is a concept of uniformity in the image. | $\sum_{i,j} \left( \left( i-\mu\right)+\left( j-\mu\right) \right)^{3}g\left( i,j \right)$ |
| **Cluster Prominence** | characterizes the propensity of clustering of the pixels in the ROI. It measures the asymmetry of the GLCM | $\sum_{i,j} \left( \left( i-\mu\right)+\left( j-\mu\right) \right)^{4}g\left( i,j \right)$ |
| **Sum Avg** | measures the mean of the gray level sum distribution of an image | $\sum_{i=2}^{2Ng} igx+y\left( i \right)$ |

where g, μ, and σ are the co-occurrence matrix, the mean and standard deviations of sums (x: row, y: column), respectively.

**Table S.2.** Comparison of volumes in GTV_PET_, GTV_RFM-entropy_, GTV_RFM-contrast,_ and GTV_RFM-H-correlation_ in the heterogeneous tumors

| Case | GTV_CT_ (cm^3^) | GTV_GS_ | GTV_RFM-Entropy_ (cm^3^) | Comparison of GTV_PET40_ and GTV_RFM-Entropy_) by MAPE | GTV_RFM-Contrast_ (cm^3^) | Comparison of GTV_PET40_ and GTV_RFM-contrast_) by MAPE | GTV_RFM -H-correlation_ | Comparison of GTV_PET40_ and GTV_RFM-contrast_) by MAPE |
| --- | --- | --- | --- | --- | --- | --- | --- | --- |
|  |  | (GTV_PET40_)_)_ |  |  |  |  | (cm^3^) |  |
|  | volume (cm^3^) | volume (cm^3^) | volume (cm^3^) | Avg=10.5(%) | volume (cm^3^) | Avg=11.1(%) | volume (cm^3^) | Avg=10.5(%) |
| 1 | 299.7 | 105.2 | 121.6 | 15.6 | 118.5 | 12.6 | 120.7 | 14.7 |
| 2 | 29.2 | 17.5 | 19.4 | 10.8 | 18.4 | 5 | 17.9 | 2.2 |
| 3 | 64.5 | 16.6 | 17.7 | 6.6 | 17.9 | 7.8 | 18.5 | 11.4 |
| 4 | 99.6 | 17.4 | 19.8 | 13.7 | 20.7 | 18.9 | 19.2 | 10.3 |
| 5 | 76.2 | 29.4 | 32.3 | 9.7 | 33.11 | 12.4 | 33.85 | 14.9 |
| 6 | 55.4 | 34.3 | 38.9 | 13.4 | 36.5 | 6.4 | 40.6 | 18.3 |
| 7 | 142.3 | 28 | 30.1 | 7.5 | 29.8 | 6.4 | 32.73 | 2.5 |
| 8 | 20.4 | 14.2 | 16.2 | 14.3 | 16.8 | 18.5 | 13.1 | 7.7 |
| 9 | 262.2 | 50.7 | 56.3 | 11 | 59.3 | 17 | 60.7 | 19.7 |
| 10 | 16.6 | 10.2 | 11.3 | 10.7 | 11.12 | 9 | 10.8 | 7.8 |
| 11 | 19.8 | 13.6 | 14.4 | 4.3 | 14.8 | 8.8 | 14.1 | 3.6 |
| 12 | 33.7 | 25.5 | 2.7 | 8.4 | 2.4 | 4 | 2.65 | 6 |
| 13 | 29.2 | 20.4 | 21.8 | 6.8 | 22.1 | 5.7 | 19.6 | 3.9 |
| 14 | 252.1 | 131.2 | 151.4 | 15.3 | 104.7 | 20.1 | 154.5 | 17.8 |
| 15 | 115.6 | 59.4 | 65.5 | 10.2 | 68.4 | 15.1 | 69.4 | 16.9 |

**Table S.3.** Comparison of volumes in CTV_CT_ and CTV_RFM_ by MAPE (%) in the homogeneous tumors.

| **Case** | GTV_GS_ (GTV_CT_) | CTV_CT_ | CTV_RFM_-entropy in the threshold of 45% | Comparison of CTV_GS_ and CTV_RFM-entropy_ | CTV_RFM_-contrast in the threshold of 15% | Comparison of CTV_GS_ and CTV_RFM-contrast_ |
| --- | --- | --- | --- | --- | --- | --- |
|  |  |  |  |  |  |  |
|  | Volume (cm^3^) | Volume (cm^3^) | Volume (cm^3^) | MAPE(%)=5.0 | Volume (cm^3^) | MAPE(%)= 6.2 |
|  |  |  |  |  |  |  |
| 1 | 19.6 | 55.6 | 56.3 | 4.6 | 60.5 | 7.7 |
| 2 | 16.1 | 45.5 | 40.5 | 4.7 | 40.5 | 8.5 |
| 3 | 17.3 | 60.4 | 63.7 | 2.8 | 61.3 | 5.7 |
| 4 | 18.4 | 57.2 | 60.2 | 5.2 | 59.4 | 9.6 |
| 5 | 17.8 | 48.4 | 48.7 | 2.4 | 49.3 | 2.2 |
| 6 | 13.9 | 54.9 | 55.6 | 4.3 | 59.8 | 8.9 |
| 7 | 4.1 | 12 | 8.2 | 9.2 | 10.1 | 5.8 |
| 8 | 9.0 | 23.2 | 27.9 | 6.8 | 25.5 | 5.1 |
| 9 | 4.8 | 9.0 | 10.2 | 3.3 | 8.5 | 5.5 |
| 10 | 5.5 | 22.4 | 25.3 | 4.8 | 23.7 | 8.4 |
| 11 | 12.8 | 45.2 | 47.4 | 2.6 | 51.3 | 8.6 |
| 12 | 16.2 | 49.5 | 52.7 | 5.2 | 50.7 | 4.2 |
| 13 | 6.7 | 19.6 | 19.6 | 5.4 | 20.6 | 5.1 |
| 14 | 15.5 | 20.1 | 16.4 | 9.5 | 18.4 | 4.4 |
| 15 | 11 | 25.3 | 26.1 | 7.4 | 24.5 | 7.1 |
| 16 | 18.8 | 48.4 | 48.2 | 0.4 | 50.5 | 2.2 |
| 17 | 22.4 | 69.5 | 65.1 | 6.3 | 64.6 | 7.0 |

**Table S.4.** Comparison of volumes in CTV_PET_ and CTV_RFM_ (in entropy and contrast features) by MAPE (%) in the heterogeneous tumors.

| **Case** | GTV_GS (_GTV_PET40)_ | CTV_GS_ (CTV_PET40_) | CTV_RFM-entropy_ | Comparison of CTV_GS_ and CTV_RFM-entropy_ by MAPE(%) | CTV_RFM-contrast_ | Comparison of CTV_GS_ and CTV_RFM-contrast_ by MAPE(%) |
| --- | --- | --- | --- | --- | --- | --- |
|  | volume (cm^3^) | volume (cm^3^) | volume (cm^3^) | Avg =10.5 | volume (cm^3^) | Avg =9.4 |
| 1 | 105.2 | 215.3 | 248.2 | 15.2 | 239.1 | 11 |
| 2 | 17.5 | 45.2 | 48.7 | 7.7 | 52.1 | 15.2 |
| 3 | 16.6 | 42.3 | 48.6 | 14.8 | 45.6 | 7.8 |
| 4 | 17.4 | 30.5 | 34.2 | 12.1 | 33.7 | 10.4 |
| 5 | 29.4 | 51.2 | 58.2 | 13.6 | 59.1 | 15.4 |
| 6 | 34.3 | 54.2 | 60.3 | 11.2 | 59.3 | 9.4 |
| 7 | 28 | 48.3 | 55.6 | 15.1 | 57.4 | 18.8 |
| 8 | 14.2 | 24.3 | 26.2 | 7.8 | 25.1 | 3.2 |
| 9 | 50.7 | 100.3 | 120.3 | 20 | 118.6 | 18.2 |
| 10 | 10.2 | 23.1 | 24.1 | 4.3 | 24.6 | 6.4 |
| 11 | 13.6 | 32.2 | 34.4 | 6.8 | 33.1 | 2.8 |
| 12 | 25.5 | 34.3 | 36.1 | 5.2 | 33.1 | 3.4 |
| 13 | 20.4 | 28.6 | 26.9 | 5.9 | 29.6 | 3.5 |
| 14 | 131.2 | 215 | 193.3 | 10 | 200.3 | 7.3 |
| 15 | 59.4 | 145.3 | 157.7 | 7.8 | 158.6 | 9.1 |
